# E2 multimeric scaffold for vaccine formulation: immune response by intranasal delivery and transcriptome profile of E2-pulsed dendritic cells

**DOI:** 10.1186/s12866-016-0772-x

**Published:** 2016-07-16

**Authors:** Maria Trovato, Francesco Maurano, Luciana D’Apice, Valerio Costa, Rossella Sartorius, Fausta Cuccaro, Sean P. McBurney, Shelly J. Krebs, Antonella Prisco, Alfredo Ciccodicola, Mauro Rossi, Nancy L. Haigwood, Piergiuseppe De Berardinis

**Affiliations:** Institute of Protein Biochemistry, C.N.R, Via Pietro Castellino 111, Naples, 80131 Italy; Institute of Food Sciences, C.N.R, Via Roma 64, Avellino, 83100 Italy; Institute of Genetics and Biophysics A. Buzzati-Traverso, C.N.R, Via Pietro Castellino 111, Naples, 80131 Italy; Division of Pathobiology and Immunology, Oregon National Primate Research Center, Oregon Health & Science University, 505 NW 185th Avenue, Beaverton, OR 97006 USA; Department of Science and Technology, University of Naples “Parthenope”, Centro Direzionale Site island C4, Naples, 80143 Italy

**Keywords:** E2 scaffold, Vaccine, Mucosal immunity, Bone marrow-derived dendritic cells, RNA-Sequencing, Transcriptome profile, Th2

## Abstract

**Background:**

The E2 multimeric scaffold represents a powerful delivery system able to elicit robust humoral and cellular immune responses upon systemic administrations. Here recombinant E2 scaffold displaying the third variable loop of HIV-1 Envelope gp120 glycoprotein was administered *via* mucosa, and the mucosal and systemic immune responses were analysed. To gain further insights into the molecular mechanisms that orchestrate the immune response upon E2 vaccination, we analysed the transcriptome profile of dendritic cells (DCs) exposed to the E2 scaffold with the aim to define a specific gene expression signature for E2-primed immune responses.

**Results:**

The in vivo immunogenicity and the potential of E2 scaffold as a mucosal vaccine candidate were investigated in BALB/c mice vaccinated *via* the intranasal route. Fecal and systemic antigen-specific IgA antibodies, cytokine-producing CD4^+^ and CD8^+^ cells were induced assessing the immunogenicity of E2 particles *via* intranasal administration. The cytokine analysis identified a mixed T-helper cell response, while the systemic antibody response showed a prevalence of IgG1 isotype indicative of a polarized Th2-type immune response. RNA-Sequencing analysis revealed that E2 scaffold up-regulates in DCs transcriptional regulators of the Th2-polarizing cell response, defining a type 2 DC transcriptomic signature.

**Conclusions:**

The current study provides experimental evidence to the possible application of E2 scaffold as antigen delivery system for mucosal immunization and taking advantages of genome-wide approach dissects the type of response induced by E2 particles.

## Background

The E2 system is a delivery vehicle in which antigenic determinants are inserted on the surface of an icosahedral scaffold formed by the acyltrasferase component (E2 protein) of the multienzyme pyruvate dehydrogenase (PDH) complex from *Geobacillus stearothermophilus* [1, 2]. E2 naturally serves as a docking unit for other large PDH subunits. This property makes it an excellent scaffold for the presentation of N-terminal fused heterologous antigens. The scaffold can be refolded in vitro to produce pure or chimeric particles similar to virions in size and complexity, it is able to confer high immunogenicity to the displayed determinants, and it is suitable for vaccine formulations [3–6]. Various protein domains, such as the HIV-1 Envelope (Env) V3 loop, can be assembled into lipopolysaccharide (LPS)-free E2 recombinant vaccines, and we previously demonstrated that their systemic administrations are able to elicit potent binding antibodies and T-cell responses in mice, as well as autologous neutralizing antibodies in rabbits [7].

A shared feature of many pathogens is that the infection occurs or initiates at a mucosal surface. While systemic vaccination offers protection against pathogens such as polio and influenza viruses, induction of mucosal immunity is required for effective protection against pathogens such as HIV, human papillomavirus, herpes viruses, *Vibrio cholera* and the *Mycobacterium* species [8–13]. Antibodies patrolling the mucosal epithelium appear to play a crucial role in blocking HIV-1 mucosal challenge [14]. Therefore, it is necessary to develop adequate mucosal vaccination protocols for this type of infection.

Understanding the immunological mechanisms of vaccination is of paramount importance for the rationale design of a vaccine. Recently, the use of systems biology approaches gave an important contribution to elucidate the fundamental mechanisms by which the innate immune system orchestrates protective immune responses triggered by vaccination [15–17]. High-throughput sequencing can be applied to explore cell transcriptome and to identify differences in gene expression and alternative splicing. In vaccinology, gene expression patterns induced by priming antigen presenting cells (APCs) could be used to predict antigen immunogenicity and the type of T-helper cell polarization, and/or help to select the appropriate adjuvant to administer in the vaccine formulation.

As proof-of-concept, here we report the immunogenicity of E2 scaffold in a model of mucosal vaccination, and show that intranasal administration of E2-based vaccines is able to induce mucosal and systemic immune responses. Through RNA-Sequencing (RNA-Seq) analysis, we attempt to identify the molecular signatures that could account for E2-primed immune responses, and analyze the gene expression profile of bone marrow-derived dendritic cells (BMDCs) pulsed with E2 scaffold compared to un-pulsed cells.

## Methods

### Purification of HIV-1 E2-based multimeric scaffolds

Expression and purification of E2 wild type (E2wt) and recombinant E2 multimeric particles displaying the HIV-1 SF162 Envelope V3 loop peptide [amino acid 291–336, reference strain HIV-HXB2; (Env(V3)-E2)], were performed following previously described protocols [7, 18]. The purified 1.5MDa E2 60-mer particles were subjected to endotoxin (LPS) removal by Triton X-114 (Sigma Aldrich, Milan, Italy) phase separation [19] and to detergent removal by using Thermo Scientific Pierce Detergent Removal Resin (Thermo Fisher Scientific, IL, USA). The final particles were tested for endotoxin using the Limulus Amebocyte Lysate (LAL) Assay (QCL-1000, Lonza, Basel, Switzerland), according to the manufacturer’s instructions and were less than 0.05EU/ml.

### Antibodies, reagents and synthetic peptides

For flow cytometric analysis, fluorescein isothiocyanate (FITC)-conjugated anti-mouse CD8 (clone 53-6.7) and phycoerythrin (PE)-conjugated anti-mouse IFN-γ (XMG1.2) were purchased from Biolegend (San Diego, CA). For Intracellular Cytokine Staining (ICS) assay, the H-2d MHC class I-restricted synthetic IGPGRAFYA_(311–318)_ peptide, corresponding to residues 311-318 of the HIV-1 Env V3 loop, was purchased from PRIMM srl (Naples, Italy). For analysis of fecal IgA, serum IgA and IgG, the HIV-1 SF162 clade B V3 peptide (PNNNTRKSITIGPGRAFYATGD) and the V3 scrambled peptide (PNNNTRKSIFYRGAPGITATGD) were purchased from Invitrogen (Carlsbad, CA) and Genscript (Piscataway, NJ), respectively. Mouse IL-4 and IFN-γ ELISA MAX^TM^*Standard* SET kits were obtained from BioLegend. Mouse CD4^+^ T cell isolation kit was purchased from Miltenyi Biotec (Bergisch Gladbach, Germany). For the quantitative measurement of IgA antibodies in fecal samples, RayBio® Mouse IgA ELISA Kit was purchased from RayBiotech, Inc. (Norcross, GA, USA).

### Mucosal vaccination with E2-based vaccines

Female BALB/c (H-2d MHC) mice, 6 to 12 week-old, were purchased from Charles River Laboratory (Lecco, Italy) and housed under specific pathogen free conditions at the Animal Facility of the Institute of Food Science of C.N.R., Avellino, Italy (accreditation no. DM.161/99). Groups of five BALB/c mice were intranasally immunized with 50 μg of E2wt, Env(V3)-E2, or Env(V3)-E2 with 1 μg/mouse of cholera toxin (CT) adjuvant (Sigma Aldrich), at weekly intervals for 7 weeks. The intranasal (i.n.) administration was carried out by inoculating 20 μl of antigens to the nostril. Fecal pellets were collected at weekly intervals, weighed, homogenized at a concentration of 10 mg/ml in 1× phosphate buffered saline (PBS), 0.01 % sodium azide (Sigma Aldrich), and centrifuged at 10,000 g for 10 min to remove debris as previously described [20]; supernatants were recovered and stored frozen. One week after the final boost, mice were sacrificed to collect spleen and serum samples.

### ELISA assay for detection of IgA and IgG antibodies

Analysis of fecal IgA, serum IgA and IgG antibodies (IgG, IgG1, and IgG2a) were performed by in house ELISA as previously described [20] using the synthetic HIV-1 SF162 V3 peptide, V3 scrambled peptide, Env(V3)-E2 or E2wt proteins as antigens. Briefly, 96-well ELISA plates (MaxiSorp^TM^, NUNC, NY) were precoated with 100 μl/well of 2 μg/ml of E2 antigens or V3 synthetic peptides in 1× PBS and incubated overnight at 4 °C. Plates were washed 4 times with PBS containing 0.05 % Tween-20 (Sigma Aldrich) and blocked by adding 200 μl/well of PBS containing 2 % bovin serum albumin (Sigma Aldrich) at room temperature (RT) for 2 h. After the incubation, plates were washed 4 times with washing buffer. Two-fold serial dilutions of sera (starting at 1:100 and ending to 1:12,800) and 100 μl of soluble fecal extracts were added to the plates and incubated at RT for 2 h. All samples were tested in duplicate. After washing, plates were incubated with 1:2,000 dilution of peroxidase-conjugated rabbit anti-mouse IgA, IgG, IgG1, or IgG2a antibodies (Santa Cruz Biotechnology, Dallas, USA). Results were expressed as absorbance values after blank subtraction or as reciprocal endpoint titer of the last dilution exhibiting OD_450_ ≥ 0.12. The highest dilution tested was 1:128,000. Serum titers of <1:100 were considered negative and were reported in figures with an arbitrary titer value of 1:10. Levels of total IgA antibodies in soluble fecal extracts (100 μl of 10 mg/ml) were quantified in duplicates using the RayBio® Mouse IgA ELISA Kit, according to the manufacturer's instructions. The Env(V3)-specific IgA antibodies were normalized by dividing the specific OD_450_ readings of IgA by the total IgA concentration in ng/ml [21], measured in fecal samples at week 4 for each animal from Env(V3)-E2 and Env(V3)-E2 + CT groups.

### Detection of IL-4 and IFN-γ

Spleen cells were isolated from BALB/c mice mucosally immunized with E2wt or Env(V3)-E2 administered with CT. Splenocytes from each group were pooled and used as antigen presenting cells (APCs). Afterwards, CD4^+^ T cells were purified from pooled splenocytes using the CD4^+^ T cell isolation kit. 1 × 10^5^ CD4^+^ T cells were incubated with 1 × 10^5^ γ-irradiated APCs preloaded or not with 50 μg/ml of E2wt, or Env(V3)-E2. Cells were cultured overnight in 0.2 ml of RPMI 1640 (Lonza) medium supplemented with 50 μM 2-mercaptoethanol, 1 mM sodium pyruvate, 100 U/ml penicillin, 100 μg/ml streptomycin, and 10 % Fetal Calf Serum (FCS) (all from GIBCO, Milan, Italy). Supernatants (0.1 ml/well) were assayed in duplicate for detecting IL-4 and IFN-γ, using the mouse IL-4 and IFN-γ ELISA MAX^TM^ Standard SET kits, according to the manufacturer's instructions.

### Analysis of CD8 T-cell response

The antigen-specific CD8 T-cell response was assessed by intracellular cytokine staining (ICS) assay on spleen cells isolated from mice described above co-cultured in vitro (2.5 × 10^6^ cells/ml) for 6 days with γ-irradiated (10,000 rad) LPS-blasts (1.25 × 10^6^ cells/ml) prepulsed with 10 μg/ml of MHC-I restricted V3 peptide. IFN-γ production was evaluated on CD8^+^ gated T cells by FACS (Fluorescence Activated Cell Sorter) analysis using FACSCanto II flow-cytometer and DIVA (Data-Interpolating Variational Analysis) software (Becton Dickinson, Fullerton, CA), as previously described by Jaworski et al. [7].

### BMDCs, RNA-Seq library production, sequencing and data analysis

Bone marrow-derived dendritic cells (BMDCs) were generated according to previously described protocols [22]. Briefly, bone marrow cells were collected by flushing tibias of C57BL/6 mice (Charles River Laboratory) with complete medium (RPMI 1640, 50 μM 2-mercaptoethanol, 1 mM sodium pyruvate, 100 U/ml penicillin, 100 μg/ml streptomycin, 10 % FCS). Cells were seeded in bacteriological Petri dishes in complete medium supplemented with 200 U/ml of recombinant murine granulocyte-macrophage colony stimulating factor (GM-CSF, Peprotech, NJ, USA) for 7 days. Immature cells were harvested and incubated or not for 1 more day with 50 μg/ml of LPS-free E2wt antigen at a final concentration of 2.5 × 10^6^ cells/ml. Total RNA was extracted from untreated or E2-pulsed BMDCs using Tri Reagent (Sigma Aldrich) according to manufacturer's protocol. RNA integrity was assessed as described in Costa et al. [23]. Paired-end libraries (100 x 2 bp) prepared using TruSeq RNA Sample Preparation Kit (Illumina Inc., San Diego, CA) were sequenced on Illumina HiSeq2000 platform. About 200 million paired-end reads were sequenced. Quality was assessed using FastQC (http://www.bioinformatics.babraham.ac.uk/projects/fastqc/). TopHat version 2.0.10 [24] was used to map reads on reference mouse genome (mm9) and RefSeq mouse transcripts annotation with default parameters. More than 95 % of sequenced reads uniquely mapped to mm9. Such reads were used for further analyses. Coverage files were produced using BEDTools and loaded on UCSC Genome Browsers to analyze gene-specific features. Gene expression was measured using Cufflinks 2 [25]. Cuffdiff and CummeRBund [26] were used to identify differentially expressed genes using normalized expression values (FPKM, Fragments Per Kilobase of transcript and Million of mapped reads). An arbitrary threshold of 0.05 FDR (False Discovery Rate) and 1 FPKM in at least one condition was used to filter out differentially expressed genes. Gene ontology and pathway analysis were performed using DAVID (Database for Annotation, Visualization and Integrated Discovery) [27].

### Statistics

Statistical analyses were performed using the unpaired two-tailed Student's *t*-test. Differences were considered statistically significant when *P* < 0.05.

## Results

### Dissection of fecal antibody responses after mucosal immunization

To determine whether HIV-1 epitopes displayed on the surface of E2 scaffold could elicit antigen-specific mucosal immune responses, the V3 loop of HIV-1 Envelope gp120 glycoprotein was expressed as an N-terminal fusion to the catalytic core domain of E2 and purified according to previously described methodologies [7]. The HIV-1 clade B primary isolate SF162 (Tier 1) V3 portion (46 aminoacids total) inserted into the E2 scaffold contained the epitope (G_312_PGR_315_) recognized by the human anti-V3 monoclonal neutralizing antibody (NmAb) 447-52D and the H-2d restricted CTL (cytotoxic T lymphocyte) epitope (I_311_GPGRAFYA_319_). The resulting vaccines were nonreplicative multimeric particles formed by HIV-1 antigens inserted on the surface of E2_60-mer_ scaffold protein, and we evaluated their immunogenicity as mucosal vaccines in mice. To this end, BALB/c mice of the H-2d haplotype (*n* = 5 per group) were intranasally immunized with Env(V3)-E2, administered with or without the adjuvant cholera toxin (CT). Vaccinations were conducted weekly for seven weeks. Fecal pellets were collected after each immunization.

**Fig. 1:**
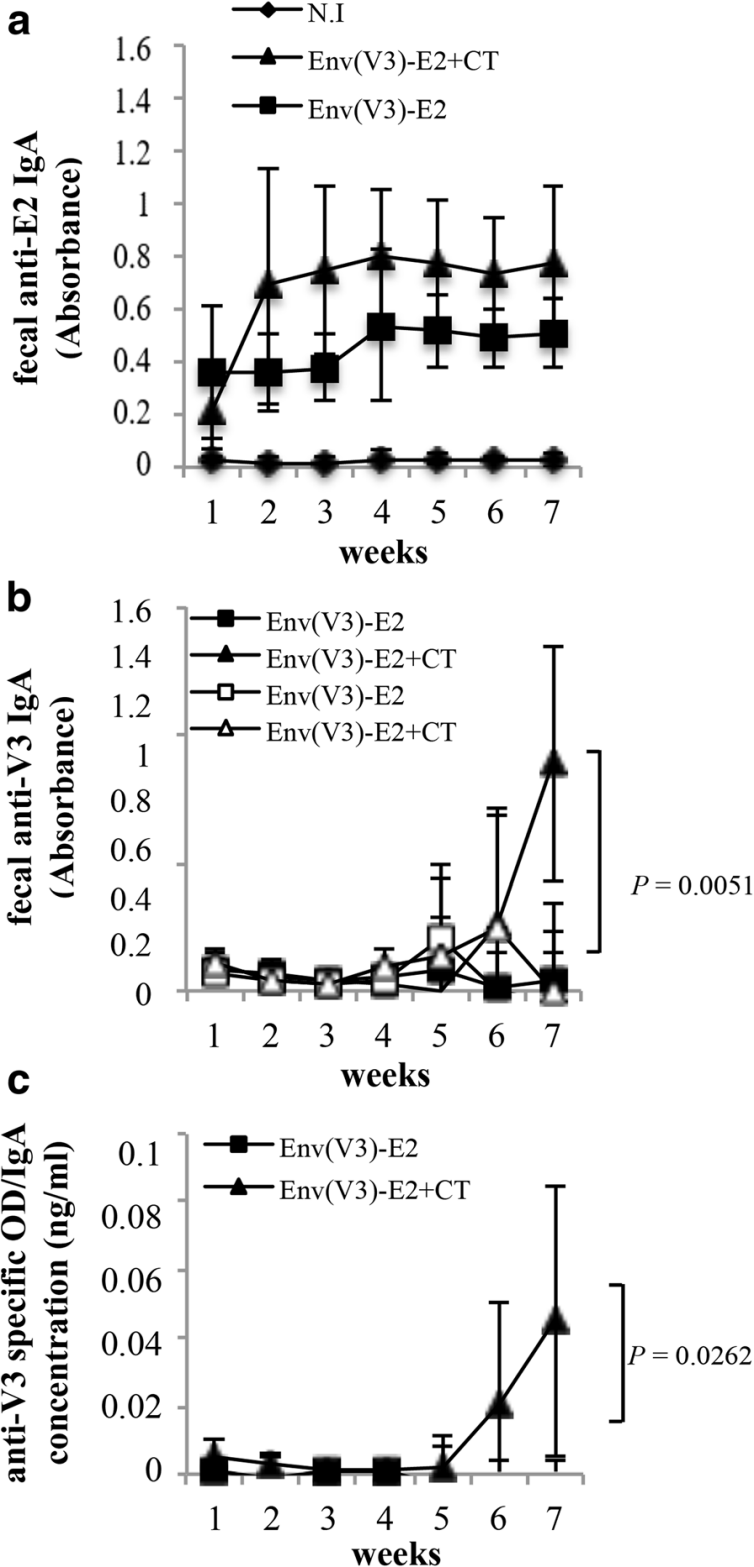
Mucosal antibody responses. BALB/c mice (*n* = 5) were intranasally immunized with: Env(V3)-E2 particles administered with (triangle) or without (square) CT adjuvant. At weekly intervals fecal pellets were collected to determine the presence of E2-binding antibodies and Env-specific IgA. Naïve mouse group (N.I., *n* = 5, diamonds) was used as control of background response. Fecal anti-E2 and anti-V3 IgA antibodies were measured by coating in ELISA assay (**a**) E2wt protein or (**b**) synthetic HIV-1 SF162 V3 peptide (filled triangles or squares), and scrambled peptide (empty triangles or squares). A representative experiment out of two is shown. Graphs show the mean absorbance values (± S.D.) of fecal samples from mice of each group at the indicated time points; statistical significance was determined using the unpaired two-tailed Student's *t*-test, *P* value is reported. (**c**) Specific levels of IgA antibodies to V3 peptide as defined by ELISA OD_450_ readings normalized to whole IgA concentration in soluble fecal extracts from mice immunized with Env(V3)-E2 particles administered with (triangle) or without (square) CT adjuvant

Mucosal IgA responses were measured against E2wt (Fig. 1a), the synthetic HIV-1 SF162 V3-specific peptide, corresponding to residues 301-322 of the V3 loop presented on the E2 surface, and V3 scrambled peptide (Fig. 1b). Figure 1a shows the mean absorbance values for antibodies obtained in feces against the E2 scaffold reaching a plateau after 4 weeks of treatment with the Env(V3)-E2 vaccines in presence or absence of CT. After the first immunization, all vaccinated mice generated anti-carrier E2-specific responses that were enhanced following subsequent administrations (Fig. 1a) and sustained throughout the course of the experiment. No statistically significant differences (*P* > 0.05) in the absorbance values of E2-binding antibodies were observed between groups of immunized mice, suggesting that E2 scaffold is also immunogenic at mucosal sites in the absence of added adjuvant. Concerning V3-specific antibody responses, Env(V3)-E2 + CT group showed at week 7 significantly higher levels of V3-binding antibodies compared to Env(V3)-E2 group (*P* = 0.0051) (Fig. 1b). This difference remains significant after normalization of the Env(V3)-specific IgA responses to whole IgA concentration (*P* = 0.0262, Fig. 1c). Thus, in contrast to antibody responses to the E2 carrier, adjuvant was essential to induce detectable antigen-specific mucosal immune responses.

Overall, this experiment demonstrates that intranasal administration of E2-based vaccines is able to induce anti-Env antigen-specific antibodies in fecal samples.

### Analysis of systemic antibody and CD4 T cell responses

**Fig. 2:**
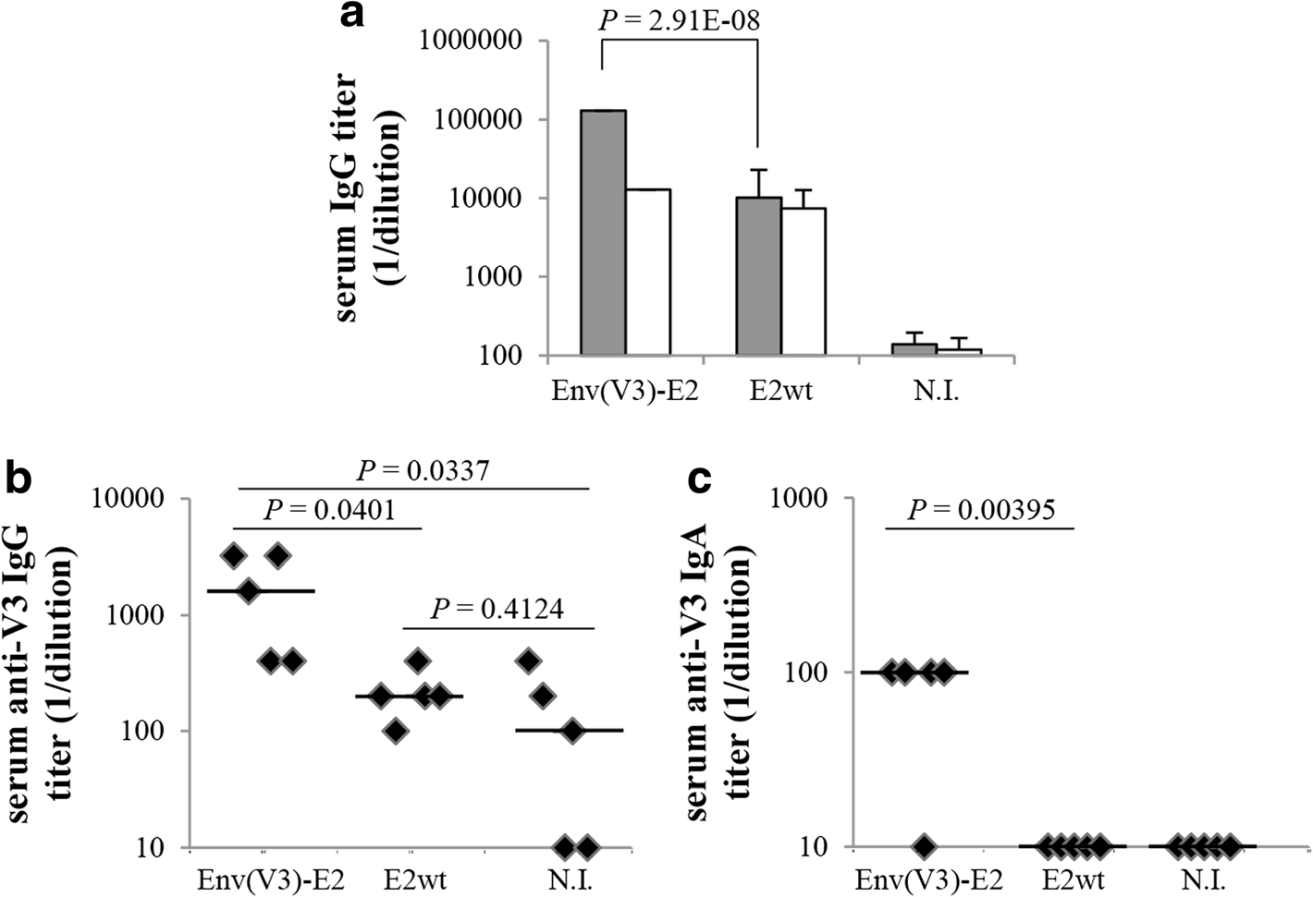
Systemic antibody responses. BALB/c mice (*n* = 5) were intranasally immunized with Env(V3)-E2 plus CT adjuvant or E2wt particles. At the end of vaccination, sera were collected to determine binding antibody responses against (**a**) E2wt (white bars), Env(V3)-E2 protein (gray bars) and (**b**-**c**) HIV-1 SF162 V3 synthetic peptide, by ELISA. Results were expressed as reciprocal endpoint titers. Naïve mouse group (N.I., *n* = 5) was used as control of background response. **a** Anti-carrier E2 and anti- Env-E2 IgG antibodies. Mean values + S.D. are shown. **b** Anti-V3 IgG and **c** anti-V3 serum IgA binding antibodies obtained from sera of each animal. Lines represent median values. *P* values determined using the unpaired two-tailed Student's *t*-test are reported. A representative experiment out of two is shown

Since the strongest V3-specific IgA response was observed following inoculation of Env(V3)-E2 administered with adjuvant, we focused our interest on this group (Env(V3)-E2 + CT) for additional experiments. The E2 wild type group (E2wt) was included to compare the responses generated against the carrier. We assessed the systemic antibody response against the E2 scaffold and the Env(V3)-E2 protein in serum samples by ELISA. All animals developed high titers (~10^4^) of anti-carrier E2 specific IgG antibodies (Fig. 2a). Mice immunized with Env(V3)-E2 particles developed significantly higher titers of Env(V3)-E2 binding antibodies compared to the E2 wild type control group (Fig. 2a). To specifically detect antibodies raised against the 447-52D core epitope we used the V3 synthetic peptide as antigen (Fig. 2b and c), and found that Env(V3)-E2 particles were able to elicit serum IgG (Fig. 2b) and IgA (Fig. 2c) V3-binding antibodies. These results indicate that intranasal delivery of E2 is able to elicit mucosal and systemic antibody responses directed toward the 447-52D core epitope of the HIV-1 V3 loop region.

In order to examine the type of immune responses induced in vivo by E2 vaccination following the intranasal route, we analyzed the isotype ratios of serum IgG1/IgG2a and evaluated the induction of antigen-specific CD4^+^ T cells in vaccinated mice.

**Fig. 3:**
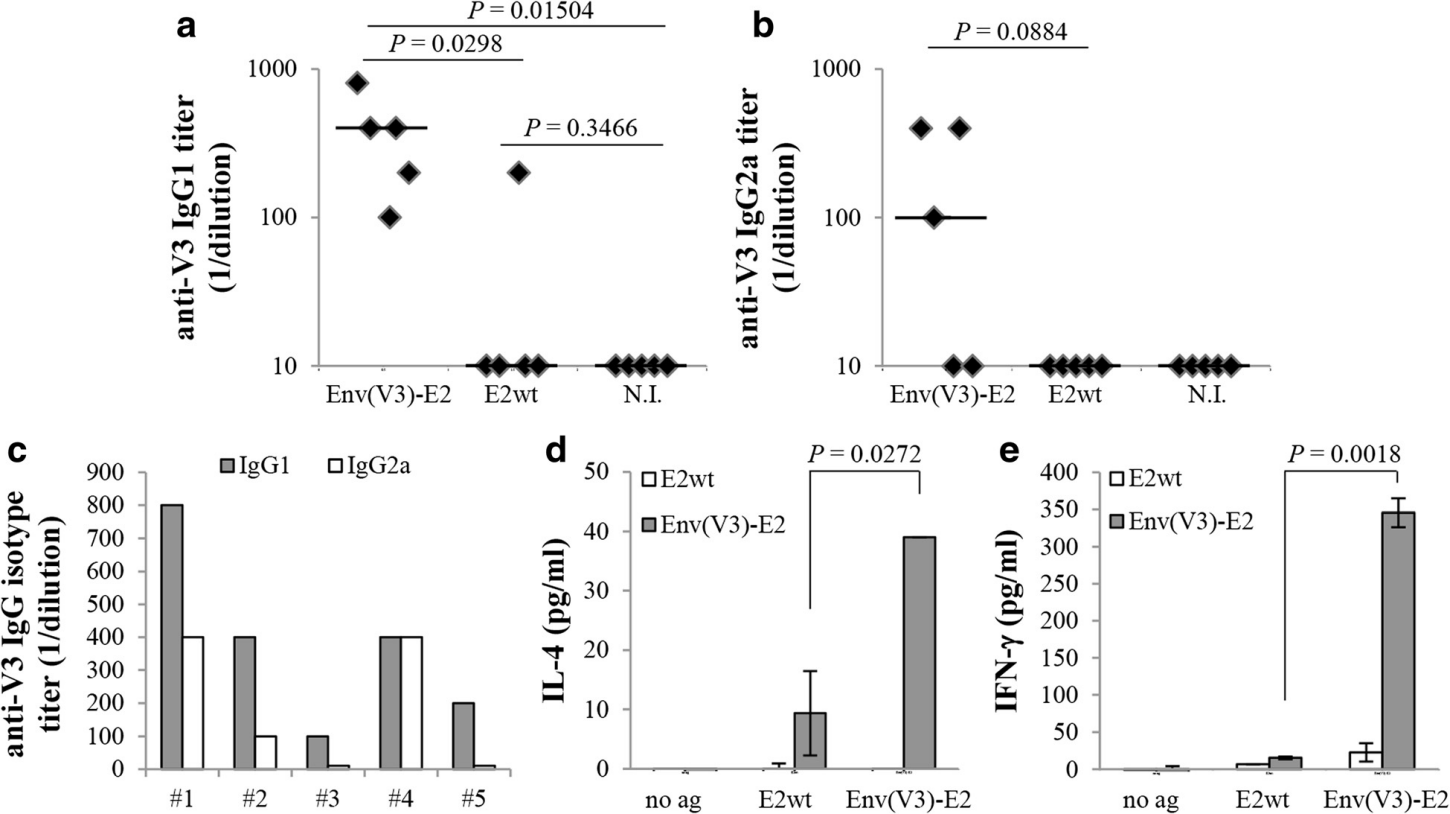
Isotype analysis and T helper cell responses. **a**-**c** Graphs show the reciprocal endpoint titers of anti-V3 isotypes from mice (*n* = 5) intranasally immunized with Env(V3)-E2 + CT or E2wt. **a** IgG1 and **b** IgG2a serum anti-V3 antibodies. Naïve mouse group (N.I., *n* = 5) was used as control of background response. **c** Anti-V3 IgG1 (gray bars) and IgG2a (white bars) endpoint titers for each animal from Env(V3)-E2 plus CT group. Lines represent median values; statistical significance was determined using the unpaired two-tailed Student's *t*-test; *P* values are reported. **d** *Ex vivo* production of IL-4 and **e** IFN-γ by purified CD4^+^ T cells isolated from mice immunized as above, in response to spleen cells pulsed with no antigen (no ag), E2wt, or Env(V3)-E2. Cytokines were detected by ELISA assay; statistical significance was determined using the unpaired two-tailed Student's *t*-test; *P* values are reported. A representative experiment out of two is shown

We first determined the isotype of anti-V3 antibodies in sera collected at the end of vaccination. Intranasal administration of Env(V3)-E2 based vaccines induced a preferential increase of IgG1 (Fig. 3a and c) against the V3 peptide over the IgG2a isotype (Fig. 3b and c), suggesting a polarization of CD4^+^ T cells toward the Th2 subset, consistent with findings previously reported by us [5, 28]. We next evaluated whether mucosal administration of Env(V3)-E2 induced Th2 cells. To this end, we characterized the type of the cellular immune response by measuring cytokine production from splenic CD4^+^ T cells. Env(V3)-E2 particles elicited antigen-specific CD4^+^ T cells able to produce the Th2 cytokine IL-4 (Fig. 3d). However, following the intranasal vaccination regimen, we also detected IFN-γ production in CD4^+^ T cells purified from vaccinated mice (Fig. 3e).

### Analysis of V3-specific CD8 T-cell response

**Fig. 4:**
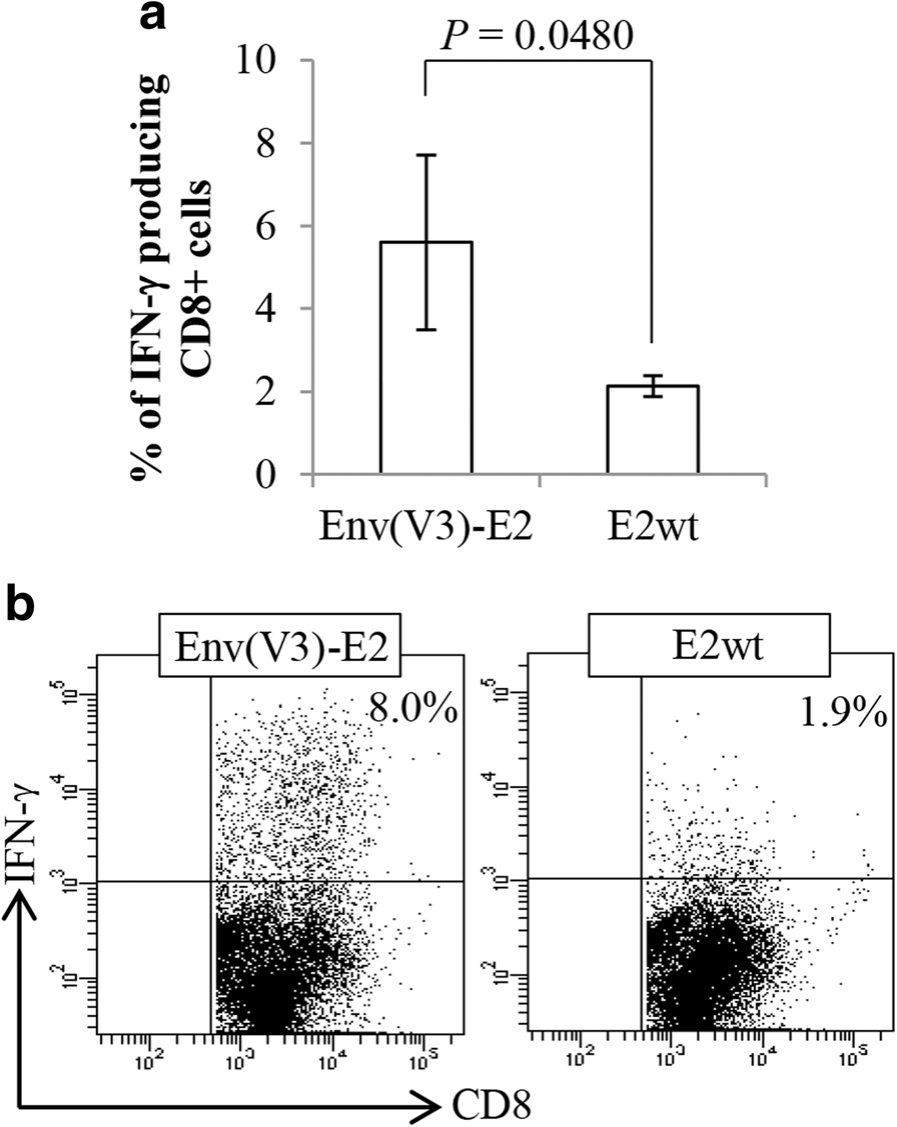
V3-specific CD8^+^ T-cell response. BALB/c mice (*n* = 3) were intranasally immunized with Env(V3)-E2 plus CT or E2wt particles. Isolated splenocytes were co-cultured with V3 (IGPGRAFYA_311-318_) peptide-pulsed γ-irradiated LPS-blasts; after 6 days of in vitro stimulation, effector cells were assayed for IFN-γ production by ICS. **a** Graph represents the mean (± S.D.) of percentage values of V3-specific CD8^+^ gated T cells secreting IFN-γ from all mice in each group. **b** Representative dot plot analysis of IFN-γ ICS from a single mouse in each group with the percentage values of IFN-γ positive cells indicated in the upper right corner of each square. Statistical significance was determined using the unpaired two-tailed Student's *t*-test; *P* value is reported. A representative experiment out of two is shown

Since CD8^+^ T cells control the replication of highly pathogenic immunodeficiency viruses [29, 30], we also investigated the antigen-specific CD8^+^ T-cell response following the intranasal route. One week after the final boost, isolated splenocytes were analysed for IFN-γ production after 6 days of in vitro stimulation with V3-pulsed LPS-blasts. In Fig. 4 we reported the percentage values of V3-specific IFN-γ producing CD8^+^ T cells (Fig. 4a) and representative dot plots of ICS (Fig. 4b). Intranasal administration of Env(V3)-E2 particles was able to induce V3-specific CD8^+^ T cells that produced IFN-γ.

### Analysis of transcriptome profile of E2-pulsed BMDCs

**Fig. 5:**
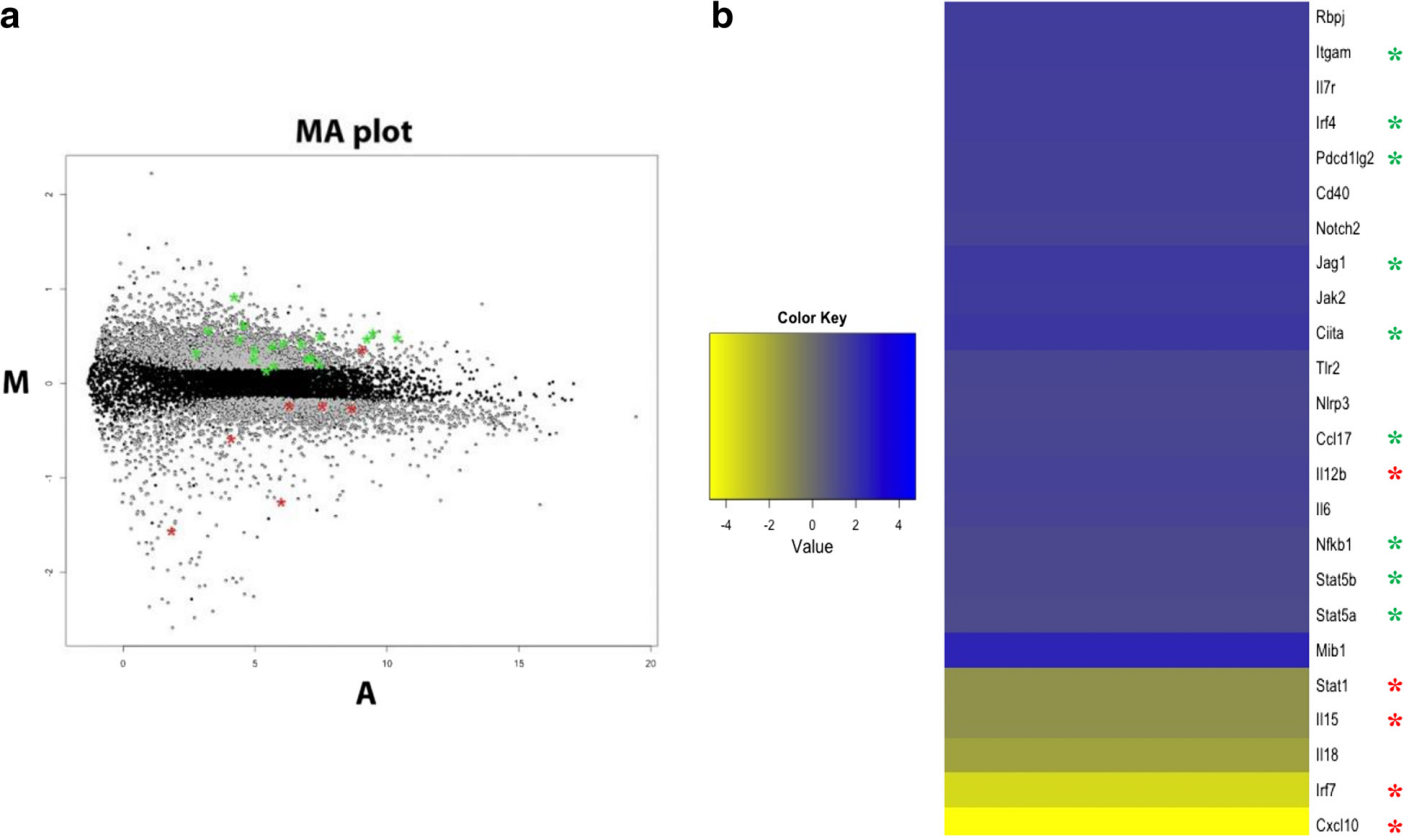
RNA-Seq analysis of E2-pulsed BMDCs. **a** Standard MA plot (“ratio intensity plot”) of the FPKM (Fragments Per Kilobase of transcript and Million of mapped reads) of E2-pulsed bone marrow-derived dendritic cells (BMDCs) *vs* untreated cells per each RefSeq gene. The y-axis represents the fold change (M = Log Ratio) and the x-axis the Average Intensity (A) for each gene (dot). Gray dots indicate differentially expressed (DE) genes. **b** Heatmap showing the fold change in gene expression for selected DE genes in E2-pulsed BMDCs *vs* untreated cells. Green asterisks include Th2 and MHC-II related genes; red asterisks include Th1-associated genes

We used genome-wide transcriptional approach to identify gene expression signatures induced in antigen presenting cells by E2 vaccine, to understand and elucidate the molecular mechanisms that account for the E2-primed immune responses. In detail, we performed RNA-Sequencing analysis on mouse immature BMDCs cultured with LPS-free E2wt particles to explore the impact of E2 scaffold on the transcriptional profile of BMDCs. Comparing the transcriptome of un-pulsed BMDCs with the transcriptome of E2-treated cells, we observed a substantial deregulation of gene expression after E2 treatment (more than 5300 genes with FDR < 0.05). In particular, a significant up-regulation was measured for 2984 genes upon E2 stimulation (Fig. 5a). Statistically significant molecular pathways perturbed in E2-pulsed BMDCs were defined by mapping up-regulated genes to KEGG (Kyoto Encyclopedia of Genes and Genomes) pathways using the Database for Annotation, Visualization and Integrated Discovery [27]. Such analysis revealed that E2 exposure triggers the up-regulation of genes associated with immune response, such as the “Chemokine signaling” and the “Jak-STAT signaling” pathways, and the expression levels of selected genes involved in these pathways are illustrated in Fig. 5b. Interestingly, RNA-Seq data revealed a significant up-regulation of genes correlated with type-2 polarized DCs (green asterisks in Fig. 5a and b). Among them, a significant transcriptional activation was measured for genes encoding Janus Kinase 2 (*Jak2* gene), the Signal Transducer and Activator of Transcription 5, STAT5 (*Stat5a* and *Stat5b* genes), the transcription factor Nf-kB (*Nfkb1*), the Th2 chemokine CCL17 (*Ccl17* gene), the Notch ligand Jagged-1 protein (*Jag1* gene*)* and Interferon regulatory factor 4 (*Irf4* gene). Notably, an opposite expression trend was observed for genes encoding proteins related to Th1 cell polarization. In particular, interferon signaling genes such as *Stat1* and *Irf7*, and chemokine and interleukin encoding genes - including *Cxcl10* and *Il15* - were significantly down-modulated after BMDC exposure to E2 compared to untreated cells (red asterisks in Fig. 5a and b). Since the expression of *Irf4* has been positively correlated to a transcriptional activation of Th2 polarization-related genes [31], we investigated the expression level of these genes. In particular, RNA-Seq analysis revealed that *Itgam* (*integrin alpha M*), *Pdcd1lg2* (*programmed cell death 1 ligand 2*), and *Ciita* (*class II*, *major histocompatibility complex, transactivator*) genes were up-regulated in DCs exposed to E2. Furthermore, in E2-pulsed BMDCs, *Il12b*, *Il6* and other genes encoding receptors of innate immune system - such as *Nlrp3* (*NOD-like receptor family*, *pyrin domain containing 3*), *Tlr2* (*toll-like receptor 2*) and *Il7r* (*interleukin 7 receptor*) genes - were up-regulated. In addition, the expression levels of genes encoding the co-stimulatory molecule CD40 and the Notch2 signaling components (*Notch2*, *Rbpj*, *Mib1*) increased when BMDCs were stimulated with E2 (Fig. 5b). It should be emphasized that the Notch2-RBPJ signaling receptor has been described to control functional differentiation of DCs and specify a unique CD11b^+^ DC subset required for efficient T cell priming [32]. These results suggest that E2 scaffold may activate DCs through innate immune receptor (PRRs, pattern recognition receptors) and provide signals that program DCs to prime Th2 cells, through the expression of a Th2-type DC signature.

## Discussion

Specific immune responses are characterized by different patterns of cytokines produced by CD4^+^ T cells, an event known as polarization of helper T cells and there can be striking differences in the type of response preferentially stimulated by different carriers and/or adjuvants. In the context of a vaccine formulation, it is important to dissect the polarization induced by the E2 carrier in order to understand how to best deliver and eventually combine immunogens.

We previously described that subcutaneous administration of E2 particles expressing the Env(V3) hypervariable region elicited a sustained immune response by inducing both antibodies and antigen specific T cells [7]. Here we report that mucosally administered Env(V3)-E2 were also able to elicit mucosal and systemic V3-specific humoral immune responses. Due to the limited sample volume obtained from mice we were not able to assess the neutralization activity of the antibodies induced by E2 immunization. However, it should be emphasized that preclinical studies of HIV-1 vaccine candidates have typically shown that neutralizing antibodies (NAbs) with a long heavy chain complementarity-determining region 3 (HCDR3) are found in non-human primate and rabbit animal models, with the latter being currently the favorite model for testing candidates at eliciting NAbs. We previously reported that our Env(V3)-E2 construct was able to induce autologous NAbs when administered subcutaneously in rabbits, with a modest level of neutralization of some Tier 1 viruses [7]. For this reason we plan to assess in future work using larger animal models the induction of neutralizing antibodies by E2 mucosal immunization.

Here we also report that intranasal administration of Env(V3)-E2 particles elicits antigen-specific splenic CD8^+^ and CD4^+^ T cells. The IgG1/IgG2a ratio in the sera and the ability of Env-E2 specific CD4^+^ T cells to produce IL-4 suggest that a Th2-type of immune response is induced in mice also after mucosal immunization. Th2-type CD4 T-cell polarization has been reported to favor mucosal IgA responses [33]. Indeed, we observed the presence of V3-specific IgA in feces and anti-V3 IgG in sera of vaccinated mice following E2 mucosal administration. Since the contribution of monomeric IgA, essentially transudated serum IgA, to total IgA in fecal samples has been previously found negligible in comparison to the mucosally secreted dimeric IgA (generally less than 0.1 %) [34], we can assume that the IgA in feces from vaccinated mice were mainly of the secretory type. However, we cannot exclude the presence of monomeric IgA. In contrast to previous results obtained by subcutaneous administration [5, 28], here, we observed the ability of antigen-specific CD4^+^ T cells to produce IFN-γ. These data indicate the presence of Th2 and Th1-polarized T cells following mucosal immunization in agreement with reports demonstrating a Th1 switch in mucosally primed mice [35]. However, from our data, we cannot exclude that the production of these two cytokines may depend on the same cells.

Using an in vitro system, we measured the ability of E2 scaffold to induce gene expression changes in antigen presenting cells. We profiled the entire transcriptome of BMDCs stimulated with the E2 scaffold, showing a significant up-regulation of genes associated with Th2-polarizing DC capability [36]. Notably, we observed a significant up-regulation of the gene encoding the Notch ligand Jagged 1 (encoded by *Jag1*). Its expression in DCs has been previously described to support Notch-mediated Th2 differentiation [37]. In addition, we reported the up-regulation of *Stat5* and *Nfkb1*, whose expression is required within DC in promoting Th2 immunity [38, 39], and of *Irf4*, known to regulate the transcription of key cytokines involved in the immune response [40]. *Irf4* expression in DC has been very recently described to play a role in the initiation of the Th2 cell response [41, 42]. Previous findings also revealed that *Irf4* expression is significantly correlated with the expression of CD11b (encoded by *Itgam*), programmed death ligand-2 (encoded by *Pdcd1lg2*) and MHC class II major histocompatibility complex transactivator (encoded by *Ciita*) [31]. In agreement with this study, we report here the up-regulation of these genes in E2-stimulated DCs.

The results obtained from the transcriptome analysis by RNA-Seq for E2-pulsed BMDCs confirm, and further strengthen, our previous observations and findings. Indeed, we have reported that subcutaneous vaccination of C57BL/6 mice with E2 polarizes the immune response towards a high IgG1/IgG2a isotype ratio and induces IL-4 producing CD4^+^ T cells, even in Th1-prone strains [28]. We also observed this type of polarization when subcutaneous administration of E2 particles was performed in the absence or in presence of various types of adjuvants, such as the strong reactogenic CFA (Freund's Complete Adjuvant) or in formulation suitable for human use, as alum (Allhidrogel) or squalen-based oil in water emulsion (Addavax) [43]. However, when we administered E2 with another carrier, namely the filamentous bacteriophage fd which we know to prompt Th1 polarization, in a priming-boosting strategy, we demonstrated a shift towards a mixed Th1/Th2 type of polarized response [28], indicating that it is also possible to modulate the Th2-type of the immune response induced by E2 administration.

E2 particles free of endotoxin, administered subcutaneously in the absence of added adjuvants, are also immunogenic, although the addition of adjuvants increases the magnitude of the response [7]. However, in this case of mucosal administration we could not detect antigen-specific immune response in the absence of adjuvant administration. Indeed, RNA-Seq analysis indicates that endotoxin-free E2 particles are able to activate in DCs pathways of immune response as the chemokine and the Jak-STAT signaling pathways. Moreover, the up-regulated expression of *Il12* and *Il6* was found, while the expression levels of genes encoding other proinflammatory cytokines did not increase upon E2 stimulation. These levels of inflammatory response may explain why E2 particles free of endotoxin are immunogenic when administered subcutaneously in the absence of added adjuvants. However, it is not sufficient to prime the immune response in the absence of adjuvants in the case of mucosal delivery.

We previously have reported that Env(V3)-E2 particles were much more immunogenic when co-administered with other systems such DNA expression vectors, encoding Env proteins, delivered intradermally (*via* Gene gun) in rabbits [7]. On the basis of these findings and of the known role played by Th2 cells in activating and enhancing B cell proliferation and humoral immune responses [44], in order to increase the mucosal immune response we plan, in future work, to test the mucosal delivery of E2 particles in combination with other strongly immunogenic delivery systems.

## Conclusions

In the current preclinical study we showed that E2 scaffold is immunogenic when administered *via* mucosal route in the presence of adjuvant and thus may represent a relevant option for immunization against pathogens whose infections occur or initiate at a mucosal surface. Moreover, by using a genome-wide transcriptional approach, we attempted to dissect the mechanisms by which DCs may orchestrate the immune response following vaccination with the E2 scaffold.

## Abbreviations

APCs, antigen presenting cells; BMDC, bone marrow-derived dendritic cell; CFA, freund’s complete adjuvant; CT, cholera toxin; CTL, cytotoxic T lymphocytes; DCs, dendritic cells; E2wt, E2 wild-type; Env, envelope; EU, endotoxin unit; FACS, fluorescent activated cell sorter; FCS, fetal calf serum; FDR, false discovery rate; FITC, fluorescein isothiocyanate; FPKM, fragments per kilobase of transcript and million mapped reads; GM-CSF, granulocyte-macrophage colony-stimulating factor; ICS, intracellular cytokine staining; IFN, interferon; Ig, immunoglobulin; IL, interleukin; IN, intranasal administration; LAL, Limulus Amebocyte Lisate; LPS, lipopolysaccharide; MHC, major histocompatibility complex; NAbs, neutralizing antibodies; OD, optical density; PBS, phosphate buffer saline; PDH, pyruvate dehydrogenase; PE, phycoerythrin; PRRs, pattern recognition receptors; RNA-Seq, RNA-Sequencing; Th, T helper
